# Sex Hormones-Mediated Modulation of Immune Checkpoints in Pregnancy and Recurrent Pregnancy Loss

**DOI:** 10.3390/ijms27031265

**Published:** 2026-01-27

**Authors:** Michał Zych, Aleksander Roszczyk, Marzenna Zakrzewska, Radosław Zagożdżon, Leszek Pączek, Filip Andrzej Dąbrowski, Monika Joanna Kniotek

**Affiliations:** 1 Department of Clinical Immunology Medical, University of Warsaw, 02-006 Warsaw, Poland; leszek.paczek@wum.edu.pl (L.P.); 2 Department of Logic and Cognitive Science, Faculty of Psychology, Adam Mickiewicz University, 61-712 Poznan, Poland; 3 Laboratory of Cellular and Genetic Therapies, Medical University of Warsaw, 02-091 Warsaw, Poland; radoslaw.zagozdzon@wum.edu.pl; 4 Department of Bioinformatics, Institute of Biochemistry and Biophysics, Polish Academy of Sciences, 02-091 Warsaw, Poland; 5 Department of Gynecology and Gynecological Oncology, Medical Centre of Postgraduate Medical Education, 01-813 Warsaw, Poland; fil.dabrowski@gmail.com

**Keywords:** immune checkpoints, recurrent pregnancy loss, sex hormones, TIGIT, PD-1, TIM-3, VISTA, LAG-3

## Abstract

Recurrent pregnancy loss (RPL) is defined as the loss of two or more pregnancies before the 22nd gestational week and affects 10–15% of clinical pregnancies. Despite extensive diagnostics, over 50% of RPL cases remain unexplained, suggesting an important role for immunological mechanisms. Sex hormones (SH) are key regulators of immune responses during pregnancy; however, their influence on immune checkpoint proteins (ICPs) is poorly understood. This study evaluated the effects of progesterone, β-estradiol, and dihydrotestosterone (DHT) on ICP expression on immune cells, including Treg, NK, NKT, TC, Th, and T cells, collected from pregnant women and patients with unexplained RPL (uRPL). Peripheral blood mononuclear cells from 20 pregnant women and 20 uRPL patients were cultured for 48 h with SH. The expression of the first generation of ICPs—PD-1 and TIM-3—and the second—LAG-3, TIGIT, and VISTA—on T, NK, and NKT cells was analyzed by the flow cytometry method. In pregnant women, SH exerted modest effects, with DHT increasing VISTA and LAG-3 expression, while progesterone and estradiol mainly upregulated LAG-3 and TIM-3 on cytotoxic cells. In contrast, uRPL immune cells showed pronounced SH sensitivity, characterized by increased TIM-3 and VISTA expression and reduced TIGIT expression, particularly after DHT stimulation. In conclusion, SH modulates ICP expression in a cell-specific manner, with stronger effects observed in uRPL patients’ lymphocytes. These findings highlight a potential role for hormonal and ICP-targeted strategies in RPL management.

## 1. Introduction

A balanced interplay between the immune and endocrine systems is essential for physiological pregnancy development. Sex hormones (SH) can regulate inflammation and the recruitment of immune cells to the decidua [1,2]. The process of development of pregnancy and its immune regulation is deeply studied due to growing incidents of recurrent pregnancy loss in healthy women of reproductive age, which also has a high impact on the growing elderly population in Western Europe [3,4].

Approximately 15–25% of clinically recognized pregnancies end in miscarriage, and when unrecognized early losses are included, the miscarriage rate may be as high as 30–60% of all pregnancies. Among those who do experience additional pregnancy losses, it is estimated that <5% of couples will experience two consecutive miscarriages, and just 1% of couples will have three or more [4].

Immune checkpoint proteins (ICPs) are molecules through which the organism maintains immune homeostasis [1,2]. Immune checkpoint molecules are negative modulators and inhibitory receptors, highly expressed on activated immune cells to prevent toxic effects, damage to surrounding tissue, and extracellular matrix degradation during inflammation. They also prevent autoimmunology development [1,2]. Thus, ICPs are being studied in cases of recurrent pregnancy loss (RPL), where the balance between inflammation and immune tolerance formation is crucial for physiological pregnancy progression [4,5]. Currently, the main role in immune regulation during pregnancy is given to regulatory T cells (Tregs). Tregs expressing CTLA-4 are able to induce dendritic cells (DCs) to produce indoleamine 2,3-dioxygenase (IDO). Indoleamine 2,3-dioxygenase is the key metabolic enzyme responsible for tryptophan degradation via the kynurenine pathway. IDO is widely produced in human tissues and cell subsets, including at the fetal–maternal interface, where it modulates the function of immune cells by increasing tolerogenic capacities [6]. Placental and decidual IDO protein levels correlate positively with peripheral blood Treg expression [6,7]. The enzyme is necessary to promote fetal tolerance, particularly in early gestation [7]. Moreover, it has been established that LAG-3^+^ Tregs impair effector T-cell proliferation, especially after re-exposure to fetal antigens [8]. HLA-G on extravillous trophoblasts induces PD-1 expression in decidual Tregs. The PD-1/PD-L1 pathway reduces T-cell proliferation, enhances T-cell anergy, and increases Treg activity [9]. Studies have shown that TIM-3- and PD-1-positive cytotoxic T cells at the maternal–fetal interface display strong proliferation and production of Th2 cytokines, involved in building an anti-inflammatory environment for the fetus [9]. The ICPs could be increased during Hashimoto’s disease [10,11] or alloimmune stimulation, especially after a previous pregnancy with a male fetus [12]. We excluded such influence in our research by testing our patients and controls (pregnant women) for the presence of antibodies to HLA-Y and the influence of euthyroid state on ICP results.

Sex hormones regulate both the menstrual cycle and immune tolerance during pregnancy. Progesterone (P) recruits and induces the transformation of NK cells into uterine NK cells, which facilitate embryo implantation by modulating trophoblast–endometrium interactions in the formation of spiral arteries. Progesterone triggers progesterone-induced blocking factor (PIBF) production by T cells, which downregulates perforin release in NK cells, thereby diminishing NK cytotoxicity, e.g., towards the fetus. Progesterone attenuates Th1 and Th17 immune responses and concurrently promotes the expansion of Treg cells and a shift toward Th2 polarization, which favors physiological pregnancy progression [13,14].

Estradiol is the primary biologically active estrogen secreted by the ovaries during the reproductive years, while estrone, primarily secreted from the adrenal glands, increases concentrations during reproductive senescence. Estriol is solely produced by the placenta during pregnancy [14,15]. Estrogen receptors (ERs), both nuclear and membrane-bound, have been identified in various innate and adaptive immune cell types. ER signaling regulates cell proliferation factors, growth factors, cytokines (e.g., interferons, IL-6, IL-1, VEGF, amphiregulin, and TGF-β), receptors, and signaling pathways (e.g., NF-κB, STAT, TGF-β, and TNF) [5,6].

Estrogens (E) promote Treg proliferation and T-cell maturation, with high levels favoring Th2 and low levels promoting Th1/Th17 responses [15].

Androgens, including testosterone, dihydrotestosterone (DHT), and dehydroepiandrosterone (DHEA), exert significant immunomodulatory effects through androgen receptors expressed on both innate and adaptive immune cells, such as macrophages, dendritic cells, NK cells, and T and B lymphocytes [16]. Their actions are mostly anti-inflammatory and immunoregulatory. Activation of androgen receptors promotes the upregulation of IL-10, TGF-β, and FOXP3 expression, thereby supporting Treg differentiation and stability [16,17]. In the context of reproduction, androgens contribute to the maintenance of maternal–fetal tolerance by suppressing Th1 and Th17 responses and promoting Th2 and Treg polarization. DHT has been shown to reduce pro-inflammatory cytokine production (e.g., IFN-γ, TNF-α) and inhibit cytotoxic lymphocyte activation, as well as prevent excessive immune-mediated tissue injury at the maternal–fetal interface [17,18]. DHEA supplementation has been linked to improved endometrial receptivity and immune balance through enhanced Treg recruitment and IL-10 production [17]. Overall, androgens are recognized as essential hormonal modulators of immune cells and cytokine networks during pregnancy, creating an immune milieu conducive to implantation and fetal tolerance [16,17,18].

Sex hormones are also widely used in assisted reproduction. Progesterone stabilizes the endometrium, supports implantation, and maintains early pregnancy, while estradiol enhances endometrial development [15,19]. Progesterone, in different forms, administered vaginally orally, is widely used in early pregnancy bleeding, post-IVF support, and luteal phase deficiency, despite limited evidence from randomized trials supporting its efficacy [15,19]. Androgens such as dehydroepiandrosterone (DHEA) are being investigated as adjuvants in women with diminished ovarian reserve to improve follicular development and ovarian responsiveness [17,18].

The influence of SH on immune cells’ functions is widely studied, but there is no data on its influence on ICPs. The current study aims to investigate sex hormones’ impact on ICP expression on lymphocytes during healthy pregnancy (pregnant controls) and miscarriage, in women with a history of uRPL. To our knowledge, it is the first study of the SH effect on ICP expression patterns on important immune cells creating maternal tolerance. The added value of the research is the timing of the study. We studied SH influence on ICPs of peripheral blood lymphocytes derived from healthy women with physiological pregnancy (up to 13 weeks) and women in the immediate postpartum period following a miscarriage (up to 72 h after miscarriage during the first 13 weeks of pregnancy).

## 2. Results

The studied groups, healthy pregnant and RPL women (until 72 h after miscarriage), did not differ in terms of age, internal medicine status, use of supplementary diet, or thyroid function. Additionally, statistical analysis of ICPs and euthyroid state was performed with results above *p* > 0.05, as shown in Table 1. The absence of differences in anti-HLA antibodies between the groups indicates that the observed immunological alterations were not attributable to alloimmune sensitization. The groups differ significantly only in terms of the number of miscarriages (Table 1).

### 2.1. Analysis of Differences in Expression of ICPs on Immune Cells, Including Treg, CD4, CD8, CD3, NK, and NKT Cells, Between Pregnant Women and uRPL Patients

**Figure 1:**
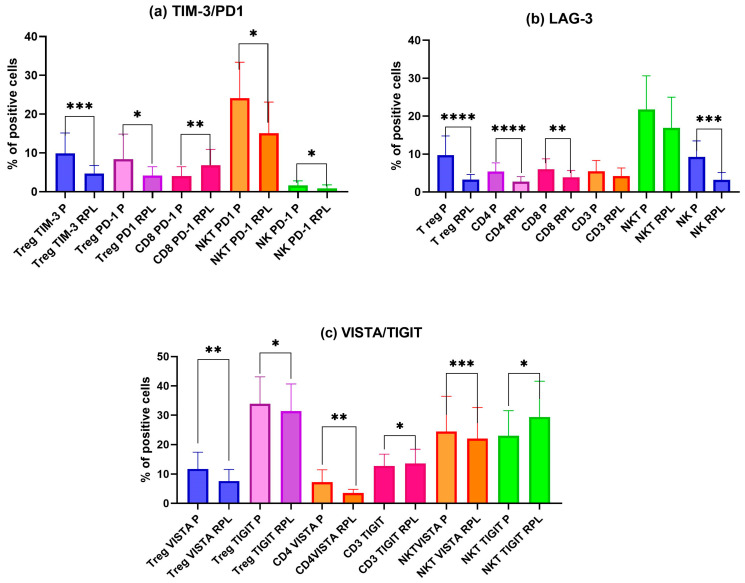
Differences in immune checkpoint protein (ICP) expression on lymphocyte subsets, including Treg, CD4, CD8, CD3, NK, and NKT cells, between pregnant (P) and RPL women, without hormonal stimulation after 48 h culture. (**a**) Significant differences in expression of TIM-3 and PD-1 on pregnant (P) and RPL women’s lymphocytes. (**b**) Significant differences in expression of LAG-3 on pregnant (P) and RPL women’s lymphocytes. (**c**) Significant differences in expression of VISTA and TIGIT in pregnant (P) and uRPL women’s lymphocytes. Data are presented as mean ± SD; * *p* < 0.05, ** *p* < 0.01, *** *p* < 0.001, **** *p* < 0.0001; P—pregnant women’s lymphocytes n = 20; RPL—recurrent pregnancy loss women’s lymphocytes. n = 20. Data statistically significant are shown for easier graph analysis.

Firstly, we analyzed the expression of ICPs in non-stimulated 48 h PBMC cultures established from pregnant and uRPL patients’ lymphocytes (n = 20 per group). We examined expressions of the first- and second-generation ICPs: TIM-3, PD-1, LAG-3, VISTA, and TIGIT on Treg, NK, NKT, Tc, Th, and T cells for each group. To enhance clarity and facilitate data interpretation, the data presented in Figure 1 refer to significant changes in ICPs expression; the most significant changes are not shown.

Overall, ICP expression in the studied subpopulation of RPL lymphocytes was lower than in pregnant women. The most pronounced differences were observed in Treg lymphocytes in the expression of TIM-3, PD-1, LAG-3, VISTA, and TIGIT (Figure 1a–c). NKT cells from pregnant women had evidently higher expression of PD-1, TIM-3, VISTA, and TIGIT (Figure 1a,c). NK cells showed pronounced PD-1, LAG-3, VISTA, and TIGIT expression patterns in pregnant women compared to RPL women during miscarriage (Figure 1a–c).

### 2.2. Analysis of Differences in Expression of ICPs on Pregnant Women’s Immune Cells, Including Treg, CD4, CD8, CD3, NK, and NKT, After SH Stimulation

**Figure 2:**
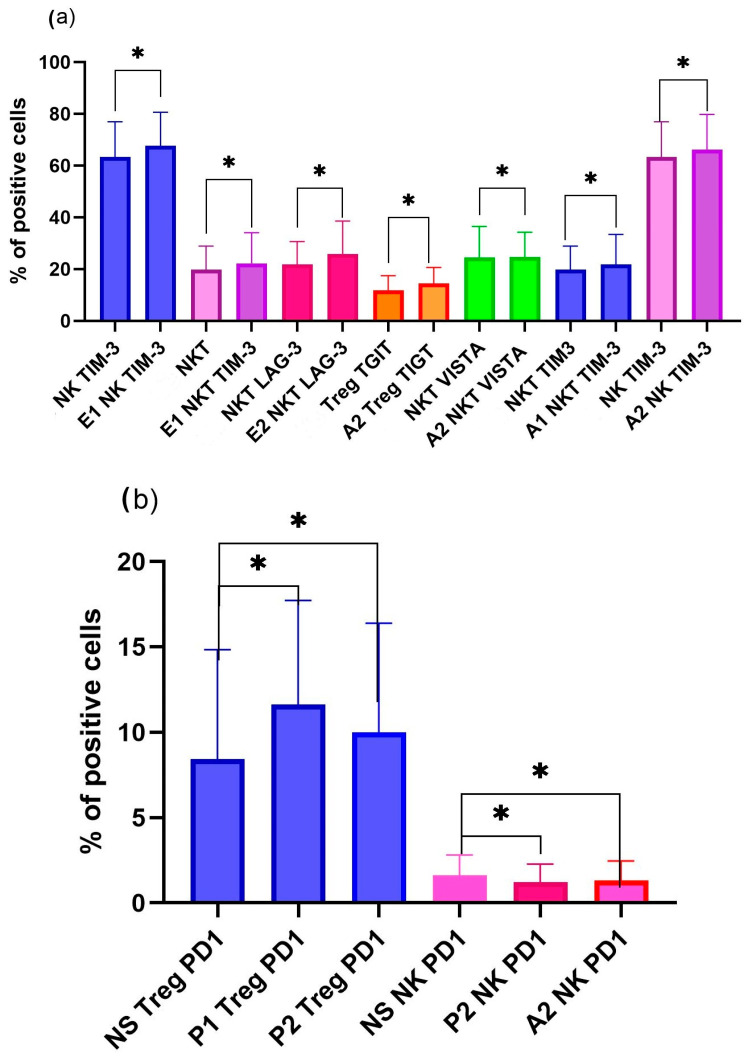
Sex hormones’ influence on the expression of ICPs on pregnant women’s lymphocyte subpopulations, including Treg, NKT, and NK cells, in 48 h in vitro cultures; graphs present only significant changes across immune cell populations. n = 20. (**a**) Influence of β-estradiol “E” and dihydrotestosterone “A” on TIM-3, LAG-3, TIGIT, and VISTA expression on NK, NKT, and Treg cells. (**b**) Influence of progesterone and dihydrotestosterone on PD-1 expression on Treg and NK cells. Number of tested pregnant women for each population and ICP, n = 20. E1—250 pg/mL β-estradiol; E2—750 pg/mL β-estradiol; A1—250 pg/mL dihydrotestosterone; A2—500 pg/mL dihydrotestosterone. Data are presented as mean ± SD of the percentage of immune checkpoint–positive cells. Statistical significance: * *p* < 0.05.

**Figure 3:**
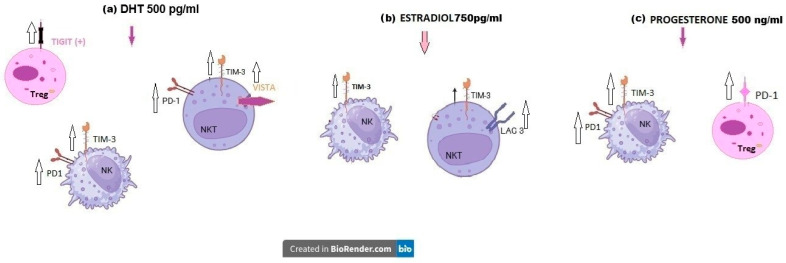
Schematic illustration of the effects of 48 h of sex-hormone treatment on immune checkpoint protein (ICP) expression in peripheral blood mononuclear cells (PBMCs) of pregnant women, n = 20, (**a**) Upregulation of TIGIT expression on Treg, PD-1, TIM-3 on NK cells, and VISTA on NKT cells after dihydrotestosterone treatment; (**b**) effect of estradiol on TIM-3 and LAG-3 expression on NKT cells and TIM-3 on NK cells of pregnant women; (**c**) effect of progesterone on TIM-3, PD-1 expression on NK, and PD-1 expression on Treg cells of pregnant women; the influence is marked with arrows, increase—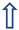 or decrease—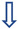. Created with Microsoft Paint 3D.

In analyzing hormonal stimulation, we noted a weaker effect of SH on ICPS in the 48 h PBMC cultures derived from pregnant women than in those from RPL patients. Only minor changes in the expression of immune checkpoint molecules were observed in the control group, whereas lymphocytes from RPL patients showed a more pronounced response to hormonal treatment, suggesting a higher sensitivity or dysregulation of hormone-dependent immune pathways during miscarriage (Figure 2 and Figure 3). In general, cells with cytotoxic properties, such as NK, NKT, and cytotoxic T cells, as well as regulatory T cells (Tregs), appeared to be more responsive to hormonal stimulation.

Sex-hormone treatment affected the expression only of several immune checkpoint receptors in lymphocyte subpopulations from pregnant women (Figure 2). Progesterone (in both concentrations of P1 and P2) markedly increased the proportion of PD-1-positive Treg cells compared with non-stimulated cells (NS). PD-1-positive NK cells showed the opposite trend: exposure to progesterone (P2) and dihydrotestosterone (A2) resulted in a significant decrease in PD-1 expression (Figure 2b).

β-estradiol in both concentrations (E1—250 pg/mL, E2—750 pg/mL) as well as androgens (A1—250 pg/mL, A2—500 pg/mL) significantly increased the proportion of TIM-3-positive NK and NKT cells of pregnant women. LAG-3 expression on NKT cells was also elevated following estradiol (E2) stimulation. Dihydrotestosterone (A2) enhanced the frequency of TIGIT-positive Treg cells and VISTA-positive NKT cells (Figure 2a).

To summarize the observed effects, a schematic presentation of hormone-dependent modulation of immune checkpoint protein (ICP) expression in pregnant women’s lymphocytes is shown in Figure 3. The diagram illustrates distinct patterns of ICP regulation in peripheral blood lymphocytes from pregnant women following 48 h of hormone stimulation (Figure 3).

### 2.3. Analysis of Differences in Expression of ICPs on uRPL Women’s Immune Cells, Including Treg, CD4, CD8, CD3, NK, and NKT

#### 2.3.1. Influence of Dihydrotestosterone on ICPs of uRPL Women’s Lymphocytes

**Figure 4:**
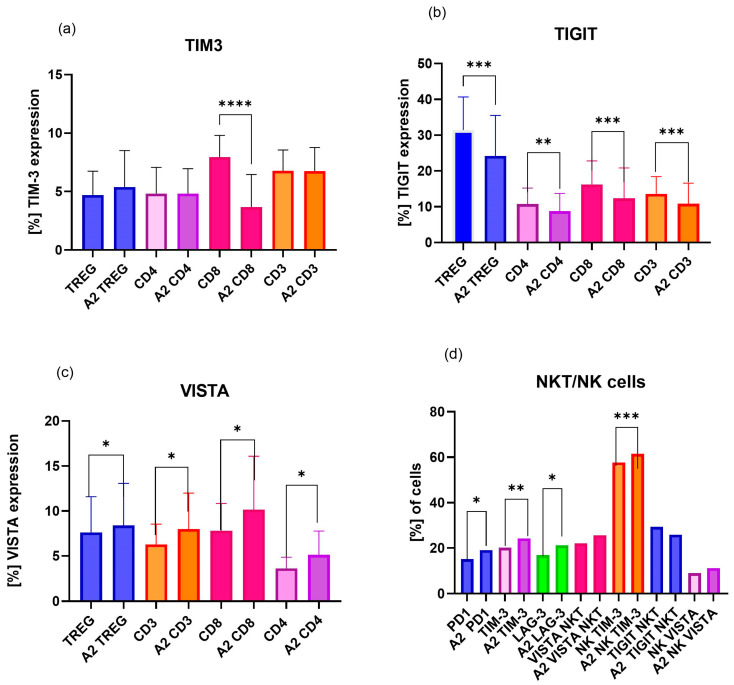
Effects of dihydrotestosterone (DHT) 500 pg/mL (A2) stimulation on immune checkpoint protein (ICP) expression on immune cell subpopulations, including Treg, CD4, CD8, NKT, and NK cells, of uRPL women, n = 20; (**a**) TIM-3, (**b**) TIGIT, (**c**) VISTA, (**d**) PD-1, LAG-3, VISTA, and TIGIT expression on NKT and NK cells. TIM-3 is highly expressed on NK and NKT cells; therefore, some results were combined for a clearer presentation of the data, graph (**d**). Data are presented as mean and SD of the percentage of ICP-positive cells. Statistical significance: * *p* < 0.05, ** *p* < 0.01, *** *p* < 0.001, **** *p* < 0.0001.

Taking into account the results regarding the effects of hormones on uRPL patients’ lymphocytes, particular changes were noted. DHT powerfully downregulated TIM-3 on CD8-Tc cells with *p* = 0.000001 (Figure 4a), and simultaneously upregulated positive TIM-3NK (*p* = 0.0010) and NKT-cell (*p* = 0.0062) populations (Figure 4d). A tremendous impact of DHT at high concentration (A2—500 pg/mL) on TIGIT, an inhibitor of cytotoxicity, was noted. DHT significantly impaired TIGIT expression on T regulatory cells, canonical T cells, and subpopulation Th dan Tc cells, as well as NK and NKT cells (Treg *p* = 0.0009; CD4 *p* = 0.0038; CD8 *p* = 0.0005; CD3 *p* = 0.0002; NKT *p* = 0.0161; NK = 0.0076) (Figure 4b).

An analogous effect of DHT on VISTA-positive immune cell populations was noted. DHT induced upregulation of VISTA expression in all studied cell populations, with “*p*” values ranging between 0.001 and 0.005 (Figure 4c), except for changes in Treg, which did not reach statistical significance.

Noteworthy observations of ICPs’ appearance were made by examining NKT cells (Figure 4d). Namely, the significant upregulation of all studied ICPs was observed on NKT cells—PD-1 *p* = 0.0150, TIM3 *p* = 0.0062, LAG-3 *p* = 0.0018, VISTA *p* = 0.0215—after DHT stimulation (Figure 4d).

#### 2.3.2. Influence of β-Estradiol on ICPs of uRPL Women’s Lymphocytes

**Figure 5:**
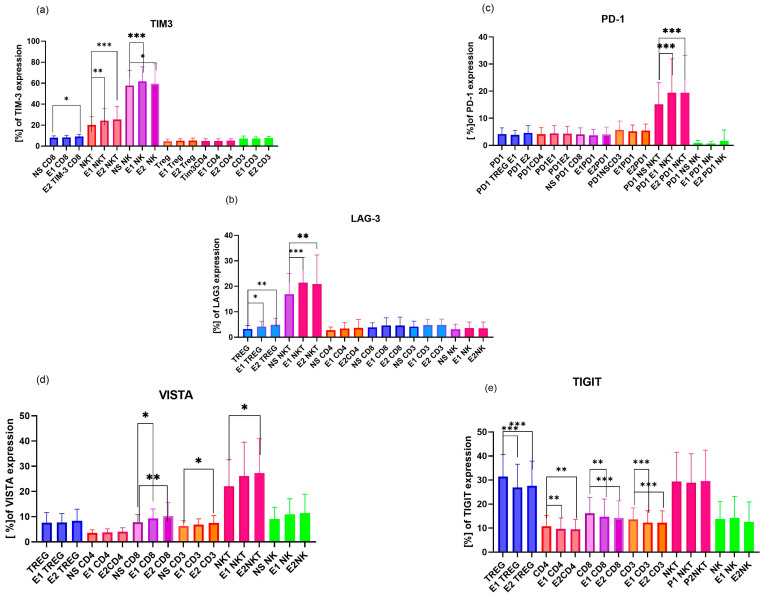
Effect of β-estradiol at concentrations of E1—250 pg/mL and E2—750 pg/mL on immune checkpoint protein (ICP) expression on immune cell subpopulations, including Treg, CD4-Th, CD8-Tc, NKT, and NK cells, of uRPL women, n = 20. (**a**) TIM-3 expression, (**b**) LAG-3 expression, (**c**) PD-1 expression, (**d**) VISTA expression, and (**e**) TIGIT expression. Data are presented as the mean and SD of the percentage of ICP-positive cells. Statistical significance: * *p* < 0.05, ** *p* < 0.01, *** *p* < 0.001; E1—250 pg/mL, E2—750 pg/mL.

Evaluation of β-estradiol impact on ICPs demonstrated that both tested concentrations (E1: 250 pg/mL and E2: 750 pg/mL) produced comparable effects across the analyzed immune cell populations. In particular, TIM-3 expression was significantly upregulated on cytotoxic cell subsets, including Tc cells (E1: *p* < 0.0001), NKT cells (E1: *p* = 0.0002; E2: *p* = 0.0005), and NK cells (E1: *p* = 0.0001; E2: *p* = 0.0136) (Figure 5a).

Both estradiol concentrations enhanced LAG-3 expression on regulatory T cells (E1: *p* = 0.0389; E2: *p* = 0.0016) and NKT cells (E1: *p* = 0.0001; E2: *p* = 0.0074), without affecting the proportion of LAG-3-positive cells in other analyzed populations (Figure 5b).

TIGIT expression was consistently downregulated by β-estradiol across canonical T-cell and regulatory T-cell populations, with no significant changes observed in NK or NKT cells (Figure 5c).

A significant increase in the proportion of VISTA-positive cells was observed exclusively at the higher estradiol concentration (E2: 750 pg/mL) in cytotoxic Tc cells, total T cells, and NKT cells (Figure 5d).

Notably, programmed death protein 1 (PD-1) expression did not exhibit significant changes across T, NKT, or NK cell populations following estradiol stimulation at either concentration.

#### 2.3.3. Influence of Progesterone on ICPs of uRPL Women’s Lymphocytes

**Figure 6:**
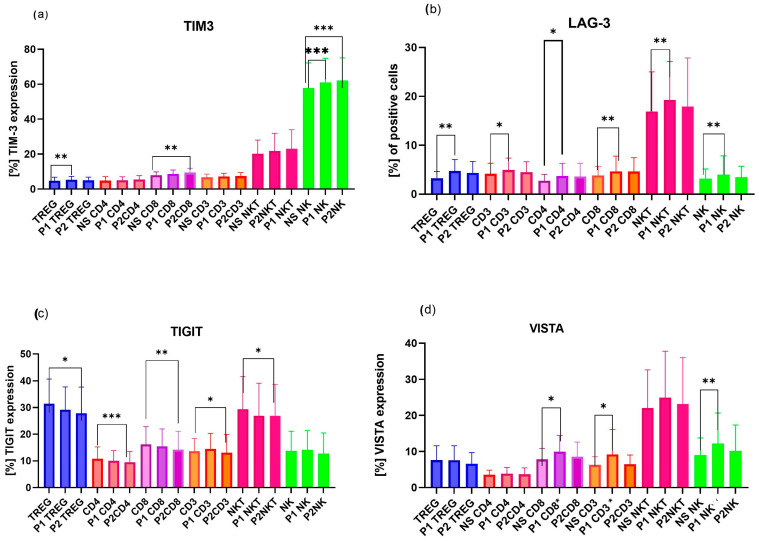
Effect of progesterone at concentrations of P1—250 ng/mL and P2—500 ng/mL on immune checkpoint protein (ICP) expression in T-cell subpopulations, including Treg, CD4-Th, CD8-Tc, NKT, and NK cells of RPL patients: (**a**) expression of TIM-3, (**b**) effects on LAG3 expression, (**c**) expression of TIGIT, and (**d**) VISTA expression. Data are presented as the mean and SD of the percentage of ICP-positive cells. Statistical significance: * *p* < 0.05, ** *p* < 0.01, *** *p* < 0.001; P1—250 ng/mL, P2—500 g/mL.

A firmer effect of progesterone stimulation on PBMCs from uRPL patients was observed at the higher concentration (P2: 500 ng/mL), resulting in significantly increased TIM-3 expression on cytotoxic cell populations, including NK cells (*p* = 0.0006), NKT cells (*p* = 0.0075), and Tc cells (*p* = 0.0002) T-CD3 *p* = 0.0023 (Figure 6a).

Progesterone treatment was also associated with sustained downregulation of TIGIT expression across most analyzed lymphocyte populations in uRPL patients, including regulatory T cells (*p* = 0.0085), helper T cells (*p* = 0.001), and NKT cells (*p* = 0.049) (Figure 6b).

At the lower progesterone concentration (P1: 250 ng/mL), significant modulation of LAG-3 expression was observed in regulatory T cells (*p* = 0.0028), T cells (*p* = 0.0011), and NKT cells (*p* = 0.0016), along with increased VISTA expression on Tc (*p* = 0.039) and NK (*p* = 0.0094) cells derived from uRPL patients (Figure 6c).

Notably, progesterone stimulation did not induce significant changes in PD-1 expression across lymphocyte populations from uRPL patients (Figure S1).

The overall SH stimulation and effects on ICPs on pregnant and uRPL lymphocytes were shown on heat-maps in Supplementary data (Figures S2 and S3).

**Figure 7:**
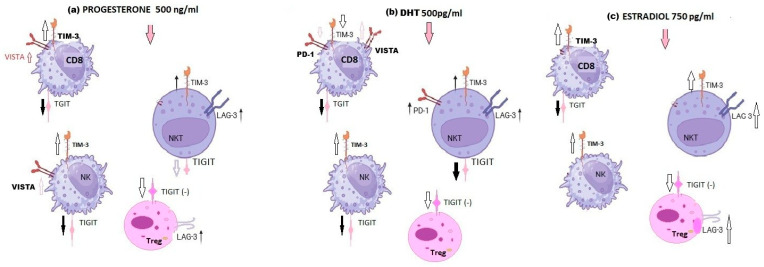
Schematic illustration of the effects of the 48 h sex-hormone culture of RPL PBMCs on immune checkpoint protein (ICP) expression: (**a**) effect of progesterone (P2, 500 ng/mL) in TIM-3, TIGIT, and LAG-3 expression on CD8^+^ cytotoxic T cells, NKT, NK, and Treg cells; (**b**) effect of dihydrotestosterone (A2, 500 pg/mL) in PD-1, TIM-3, TIGIT, and LAG-3 expression on CD8^+^ cytotoxic T cells, NKT, NK, and Treg cells; (**c**) effect of β-estradiol (E2, 750 pg/mL) on TIM-3 and LAG-3 expression in CD8^+^ cytotoxic Tc, NKT, NK, and Treg cells; the influence is marked with arrows 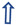 for “increase” or 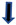 “decrease”. Created with Microsoft Paint 3D.

Overall, the upregulation by SH of ICP expression on RPL women’s lymphocytes was shown in Figure 7. All analyzed SH exert comparable effects on NKT cells, manifested by increased expression of TIM-3, LAG-3, and PD-1, together with reduced TIGIT expression, whereas the studied SH induced upregulation of TIM-3 and downregulation of TIGIT on NK cells. Regulatory T cells display decreased TIGIT expression and enhanced LAG-3 expression after SH stimulation (Figure 7).

## 3. Discussion

This study was designed to investigate the in vitro effects of the dihydrotestosterone, β-estradiol, and progesterone on immune checkpoint protein (ICP) TIM-3, PD-1, LAG-3, TIGIT, and VISTA expression on immune cells obtained from pregnant women and patients with unexplained recurrent pregnancy loss (uRPL).

Collectively, our data indicate that the expression of the analyzed immune checkpoint proteins (ICPs) is downregulated in uRPL patients compared with pregnant controls. Furthermore, ICP expression in uRPL patients exhibits heightened responsiveness to sex-hormone stimulation relative to women with physiological pregnancy, suggesting altered hormonal regulation of immune tolerance pathways. Of particular interest, dihydrotestosterone (DHT) demonstrated a pronounced effect that was synergistic with progesterone, highlighting a potential cooperative role of androgen- and progesterone-mediated signaling in the modulation of immune checkpoint regulation in uRPL patients.

Established etiological factors associated with pregnancy loss—including genetic abnormalities, endocrine dysfunctions, autoimmune diseases, anatomical abnormalities, infectious conditions, and overweight or obesity (BMI > 25)—were rigorously excluded.

The pregnant and uRPL cohorts were well matched with respect to the prevalence of diabetes mellitus, polycystic ovary syndrome (PCOS), Hashimoto’s thyroiditis, and alloimmunization. Importantly, euthyroid status in women diagnosed with Hashimoto’s thyroiditis had no measurable impact on the study outcomes.

The potential confounding effect of anti-HLA antibody presence was additionally controlled for and excluded from the analysis. Although anti-HLA antibodies are not routinely assessed in cases of recurrent pregnancy loss, they have been reported to occur in approximately 5% of pregnant women [12,20].

Anti-DDX3Y helicase antibodies were assessed in this study. DDX3Y is a member of the DEAD-box RNA helicase family and is encoded by a gene located on the Y chromosome within the azoospermia factor A (AZFa) region. Polyclonal anti-DDX3Y antibodies (catalog no. 14041-1-AP) were generated by immunization with the C-terminal region of the DDX3Y protein and were shown to specifically detect a 73-kDa band. The absence of significant differences in anti-HLA antibody prevalence between the study groups indicates that the observed alterations in immune checkpoint protein expression are unlikely to be mediated by alloantibody-dependent mechanisms. Considering the clinical comparability between the uRPL and pregnant cohorts, the differences observed in immune checkpoint protein expression are likely attributable to steroid hormone stimulation [21]. A striking observation in our study was the relatively attenuated response of PBMCs from healthy pregnant women to hormonal stimulation, which contrasts with the pronounced effects observed in lymphocytes derived from uRPL patients. This finding is not unexpected, as blood samples from uRPL patients were collected within 72 h following miscarriage, a period associated with heightened immune activation that may contribute to the observed phenomenon [22]. This discrepancy may reflect an altered hormone–immune crosstalk in uRPL and downregulation of immune checkpoint pathways during miscarriage [23,24]. Nevertheless, it is documented that the application of SH, e.g., progesterone, during miscarriage could be helpful [22,23,24,25]. In certain cases, progesterone therapy has been associated with the continuation of pregnancy; however, the characteristics of women most likely to benefit from this intervention remain unclear. Determining SH’s influence on ICP appearance in lymphocytes controlling pregnancy development may answer that question. Furthermore, hormone dosing regimens in ART differ according to the route of administration, with doses ranging from 90 mg (intravaginal) to 600 mg (oral) to 25 mg (subcutaneous) [19,20]. Thus, in our study, we tested both the lowest doses of hormones that influence ICPs in 48 h cultures of PBMCs and doses up to twice the effective dose.

Immune checkpoint proteins are widely studied in cancer therapies and are targets for immunotherapies. Similarly to cancer cells, trophoblasts express and release several ICPs like Gal-9, OX40, and PDL-1. Soluble ICPs, e.g., sHLA-G, sPDL-1, or sCTLA-4, can trigger T-cell apoptosis and Treg differentiation through retaining their receptors [26].

To date, there have been no published studies evaluating the influence of sex hormones (SH) on immune checkpoint protein (ICP) expression. Evidence from immunotherapy studies indicates that immune checkpoint protein (ICP) inhibitors can cause endocrine-related adverse effects, particularly thyroid dysfunction, which may disrupt hormone production and has been associated with impaired reproductive outcomes and fertility concerns in humans [10,11]. Thus, we challenged lymphocytes derived from pregnant and uRPL women’s peripheral blood with steroid sex hormones (SH): progesterone (250 ng/mL and 500 ng/mL), β-estradiol (250 pg/mL and 750 pg/mL), and dihydrotestosterone (250 pg/mL and 500 pg/mL).

Analysis of ICPs in non-stimulated control cultures yielded results similar to those previously published, where ICP expression was determined directly after blood collection [27]. Therefore, only results following theSH challenge will be discussed.

In general, our findings are consistent with previous reports suggesting that immune cells of RPL patients are more reactive or “primed” to endocrine modulation than healthy women’s lymphocytes [15]. Pregnant women respond weakly to higher studied concentrations of SH.

### 3.1. Dihydrotestosterone Influence on ICP Expression on Immune Cells of Healthy Pregnant Women and uRPL Patients

DHT in higher concentrations affected T reg, NK, and NKT cells of pregnant women’s lymphocytes in 48 h cultures. It improved the expression of Treg TIGIT-positive cells, TIM-3, and PD-1-positive NK cells, as well as TIM-3, VISTA, and NKT cells.

Notably, DHT was found to strongly influence the expression of ICPs (TIGIT, TIM-3, VISTA, PD-1, and LAG-3) on cytotoxic cells (Tc, NK, and NKT) of uRPL patients with miscarriage. The immunomodulatory role of DHT has been documented in several studies, highlighting its importance in maintaining immune equilibrium during pregnancy [5,9]. It was found that androgen receptors (AR) are expressed in a wide range of innate and adaptive immune cells, including neutrophils, macrophages, mast cells, monocytes, megakaryocytes, B cells, and T cells [18]. DHT exerts its effects through promoting anti-inflammatory responses via upregulation of IL-10, TGF-β, and FOXP3 expression [28,29,30]. The mechanism enhances Treg cell function and supports immune homeostasis [25]. Our observation of a boost in ICP expression on cytotoxic lymphocyte subsets may confirm previous findings on anti-inflammatory functions of DHT [31].

In our research, DHT exerted a pronounced effect on VISTA expression across nearly all analyzed lymphocyte subsets. VISTA was upregulated in Treg, CD4, and NKT cells from uRPL patients when assessed immediately after a 48h in vitro culture with 500 pg/mL DHT [32]. VISTA (V-domain Ig suppressor of T-cell activation), also known as Programmed Death-1 Homolog—PD-1H, is an immune checkpoint molecule belonging to the B7 family [30]. It functions as both a ligand and a receptor and plays a critical role in maintaining immune homeostasis and peripheral tolerance [32,33]. It delivers inhibitory signals to T cells, reducing their activation, proliferation, and cytokine production, suppresses T-cell activation and effector cytokine release (e.g., IFN-γ, TNF-α), and enhances Treg function, supporting immune tolerance. VISTA prevents excessive immune activation that could lead to tissue damage or fetal rejection [34], thus DHT may exert its suppressive function also through modulation of VISTA expression.

DHT increased the expression of TIM-3 and LAG-3, while reducing TIGIT levels on cytotoxic cells in uRPL women. These opposing regulatory effects may reflect the complex immunomodulatory activity of DHT, in which the activation of certain inhibitory pathways (TIM-3, LAG-3) coexist with the partial relief of TIGIT-mediated suppression [34,35,36]. This pattern of ICP modulation suggests that DHT may contribute to the reprogramming of Tc, NK, and NKT cells toward a more balanced or adaptive cytotoxic phenotype, rather than inducing a uniform activation or inhibition of the immune response. Functionally, this modulation could impair the cytotoxic activity of these cells while preventing excessive immune-mediated tissue damage during, for example, pregnancy complications.

Emphasized TIM-3 appearance on NK and NKT cells promotes interaction with its ligand Gal-9 [35,36]. Human trophoblast cells can induce the transformation of peripheral NK cells into a dNK-like phenotype via the secretion of galectin-9 (Gal-9) and the interaction between Gal-9 and TIM-3 [36]. In the case of unexplained miscarriage patients, a reduced rate of TIM-3 expression in dNK cells has been shown [34,35,37,38]; thus, according to our results, uRPL patients might benefit from DHT stimulation. NKT cells from uRPL patients exhibited the most distinct response to DHT across all analyzed ICPs. It was shown that peripheral blood NKT percentage is increased during pregnancy complications [36,39]. NKT cells represent a unique subset of T cells that express both T-cell and NK-cell receptors [38,39]. NKT cells are capable of rapid and abundant secretion of a broad range of cytokines, including both pro-inflammatory and anti-inflammatory mediators, enabling them to exert potent immunoregulatory effects [39]. Androgen stimulation of NKT cells resulted in increased expression of PD-1, TIM-3, LAG-3, and VISTA, which may reflect enhanced inhibitory signaling. 

Following NKT cells, cytotoxic T cells were the next population strongly influenced by DHT, exhibiting significant modulation of ICP expression. During normal pregnancy, CD8^+^ T cytotoxic cells acquire a regulatory phenotype and contribute to the establishment of maternal–fetal tolerance. These cells express immune checkpoint molecules, including PD-1, TIM-3, and LAG-3, as demonstrated in our study [40,41]. CD8^+^ T cells are also capable of secreting progesterone-induced blocking factor (PIBF) in response to progesterone signaling. PIBF exerts immunosuppressive effects by promoting Th2-type cytokine production, inhibiting NK cell cytotoxicity, and attenuating pro-inflammatory responses, thereby supporting the protection of the semi-allogeneic fetus [42]. Our present findings in the determination of TIM-3-positive CD8 cells are consistent with the results reported by Ahmadi et al. [40]. We noted substantially lower TIM-3 expression on uRPL Tc cells. DHT exposure further downregulated TIM-3 expression on Tc cells, suggesting an additional feature of hormonal modulation in immune checkpoint regulation. CD8^+^ TIM-3^+^ cells are reported to be terminally exhausted Tc cells but are able to retain functional capacity [41].

The pronounced downregulation of TIGIT by DHT, estradiol, and progesterone across all concentrations in whole studied lymphocytes from uRPL patients represents a particularly interesting observation. It was shown that TIGIT in RPL cells has lower expression on NKT and NK cells, which is in line with our results [43]. Meggyes, Matyas et al. (2022) performed a detailed characterization of NKT-cell function during pregnancy [36]. Their data demonstrate a gradual increase in TIGIT expression on NKT cells during the first trimester of healthy pregnancy, followed by continued upregulation until delivery [36].

Our observations suggest that DHT may have a more pronounced positive impact on TIGIT expression during later pregnancy (the second and third trimesters) than in early pregnancy.

An opposite effect on TIGIT expression was observed in lymphocytes from pregnant women, in which DHT upregulated TIGIT on Treg cells, TIM-3 and VISTA on NKT cells, and PD-1 on NK cells. These findings demonstrate the narrower spectrum of inhibitory effects of DHT on ICP lymphocytes from pregnant women.

### 3.2. β-Estradiol Influence on ICP Expression in Immune Cells of Healthy Pregnant Women and uRPL Patients

Lymphocytes from women with uRPL also exhibited a more prominent estradiol-mediated modulation of ICP surface expression after 48 h of culture, compared to lymphocytes from healthy pregnant women. Estradiol plays a pivotal role in the maintenance of maternal–fetal immune tolerance [43,44,45,46,47,48,49]. Elevated estradiol levels during pregnancy promote a Th2-biased immune response, thereby supporting the immunological tolerance of the semi-allogeneic fetus [44,45,46]. Estradiol modulates the activity of regulatory T cells (Tregs), natural killer (NK) cells, and cytotoxic T cells (Tc), contributing to the suppression of pro-inflammatory responses and the maintenance of a successful pregnancy [47]. The phenomenon we observed involved the modulation after estradiol stimulation of TIM-3 on Tc, NK, NKT, and LAG-3 upregulation on Treg, as well as the dampening of TIGIT on T and Treg uRPL lymphocytes. We noticed a similar effect on pregnant women’s lymphocytes, excluding Tregs. This regulatory or “silenced” state, facilitated by estradiol, may support immune homeostasis at the maternal–fetal interface and protect the semi-allogeneic fetus from immune-mediated damage [6,7,47]. LAG-3 (Lymphocyte Activation Gene-3) modulated by estradiol on NKT and Treg cells of uRPLs is an inhibitory immune checkpoint receptor functionally similar to PD-1 [49,50]. It is well documented that LAG-3-positive Tregs contribute to the maintenance of a regulatory environment during implantation [51,52]. Increased LAG-3 expression on NKT and Treg cells may reflect a shift toward an immunoregulatory or tolerogenic phenotype [53], which must be confirmed with functional studies.

### 3.3. Progesterone Influence on ICP Expression on Immune Cells of Healthy Pregnant Women and uRPL Patients

Progesterone also exerts a pronounced effect on cytotoxic lymphocytes (Tc, NKT, and NK cells) and regulatory T cells (Tregs). Similar to DHT, it induces the upregulation of TIM-3 on Tc, NKT, and NK cells, as well as LAG-3 and VISTA on T and NK cells in patients uRPL lymphocytes. In lymphocytes derived from pregnant women, progesterone induced a slight increase in PD-1 expression, restricted to Tregs and NK cells.Our results indicate that progesterone can differentially regulate multiple ICPs in both regulatory and cytotoxic immune cells of uRPL patients, potentially restoring immune balance. The selective upregulation of TIM-3 and LAG-3 on cytotoxic populations, combined with modulation of TIGIT on Treg and NKT cells, suggests a complex immunoregulatory role for progesterone that may be crucial in preventing recurrent pregnancy loss [13,14,39,42,54,55]. The increased expression of LAG-3 on cytotoxic cells such as NK, NKT, and Tc suggests reduced effector activity, limiting their ability to lyse target cells, including trophoblasts [53,54,55]. In the context of pregnancy, such modulation is considered beneficial, as it contributes to the establishment of maternal–fetal tolerance [23,34,55]. LAG-3^+^ NK, NKT, and Tc cells may acquire features of regulatory effector cells that suppress inflammatory responses and promote the secretion of immunosuppressive cytokines, including IL-10 and TGF-β [34].

DHT and progesterone significantly reduce TIGIT expression on regulatory T cells (Tregs) and cytotoxic lymphocytes, including Tc, NKT, and NK cells. Similarly, β-estradiol downregulates TIGIT expression on conventional T-cell subsets, specifically CD3^+^ and CD4^+^ T cells. The effect of sex hormones on TIGIT expression suggests that the immune system is strongly modulated by the hormonal environment [3,4,5]. The observed reduction of TIGIT on Tregs and cytotoxic cells by DHT and progesterone may lead to decreased inhibition of immune responses, potentially enhancing the effector activity of cytotoxic cells [3,5,8]. Similarly, the effect of β-estradiol on conventional T cells indicates that estrogens can modulate the balance between regulatory and effector cells, influencing immune tolerance and inflammatory responses [48,49]. Such hormonal regulation may hold particular significance in pregnancy, autoimmunity, and immunomodulatory therapies, where precise modulation of immune checkpoints is essential for maintaining immune homeostasis. Therefore, further functional investigations are warranted to elucidate lymphocyte responses following SH challenge. Taken together, our findings indicate that sex hormones influence immune checkpoint expression on lymphocytes, especially in uRPL patients. Nevertheless, hormonal stimulation did not restore expression levels to those observed in pregnant women in our research.

### 3.4. Limitations of the Study

We are aware of the limitations of the research. The hormonal stimulation experiments were performed in vitro, which may not fully reflect the physiological conditions during pregnancy or miscarriage. Only selected hormone concentrations were tested, which may not cover the full physiological range. The assessment was performed at a single time point after stimulation with SH, when we noted the greatest changes in ICP expression, so dynamic changes over time were not captured. The study focused on immune checkpoint protein expression but did not include functional assays to confirm their regulatory impact on lymphocyte activity.

## 4. Materials and Methods

### 4.1. Study Population

A total of 40 women participating in the study were investigated with respect to age, weight, height, number of miscarriages before 12 and 16 weeks of pregnancy, prodromal symptoms of pregnancy (vomiting, nausea, and breast pain), medical procedures before and during pregnancy, administration of vitamins or dietary supplements before and during pregnancy, folic acid intake 6 weeks before pregnancy, use of hormonal contraception, and fertility treatments. Additionally, data on common chronic diseases, including diabetes, endometriosis, insulin resistance, Hashimoto’s disease, and polycystic ovary syndrome, were collected and compared. Additionally, determination of anti-HLA-Y antibodies was performed to exclude immunological reasons for pregnancy loss. The data are presented in Table 1. The informed consent form for participation was distributed by the gynecologist to all participants and signed.

### 4.2. Pregnant Women

In the study, 20 pregnant women were included, each at ≤13 weeks of gestation. All participants underwent ultrasonographic examinations according to the Fetal Medicine Foundation guidelines, and blood tests were performed to confirm normal pregnancy progression.

### 4.3. Recurrent Pregnancy Loss (RPL) Women

Recurrent pregnancy loss was defined as two or more consecutive spontaneous miscarriages before 20 weeks of gestation [12]. The study group comprised 20 women experiencing subsequent pregnancy loss, before the 13th week of gestation, with all blood samples collected within 72 h after miscarriage. Women with anatomical, genetic, microbiological, immunological, or hormonal causes of miscarriage were excluded.

Detailed characteristics of the studied women are presented in Table 1.

### 4.4. Sample Isolation and Preparation

Blood was collected from the median cubital vein into heparin tubes. The isolation of lymphocytes was performed as described elsewhere [4]. After isolation, the isolated peripheral blood mononuclear cells (PBMCs) were resuspended in RPMI 1640 medium with stable glutamine (Biowest, Nuaillè, France) supplemented with 10% heat-inactivated human serum (Merck, Darmstadt, Germany), antibiotic-antimycotic solution 100 I.U., penicillin, 100 µg/mL streptomycin, and 0.25 µg/mL amphotericin (Corning, Glendale, AZ, USA). To remove hormones naturally present in human serum, the serum was incubated with dextran-coated charcoal (Merck, Darmstadt, Germany). Suspensions containing 1 × 10^6^ PBMCs per condition were cultured for 48 h in 96-well plates in the presence of the following hormones: progesterone (250 ng/mL and 500 ng/mL), β-estradiol (250 pg/mL and 750 pg/mL), and dihydrotestosterone (250 pg/mL and 500 pg/mL) (Merck, Darmstadt, Germany).

### 4.5. Flow Cytometry Staining

After culture, the expressions of PD1, TIM-3, LAG-3, TIGIT, and VISTA were analyzed on T lymphocytes-CD3^+^CD4^+^CD8^+^, T helper-CD3^+^CD4^+^CD8^−^, T cytotoxic- CD3^+^CD8^+^CD4^−^, T regulatory-CD4^+^CD25^++^CD127^low^, CD3^+^CD56^+^CD16^−^, and NK-CD3^−^ CD56^+^CD16^+^ cells with the flow cytometry method.

**Figure 8:**
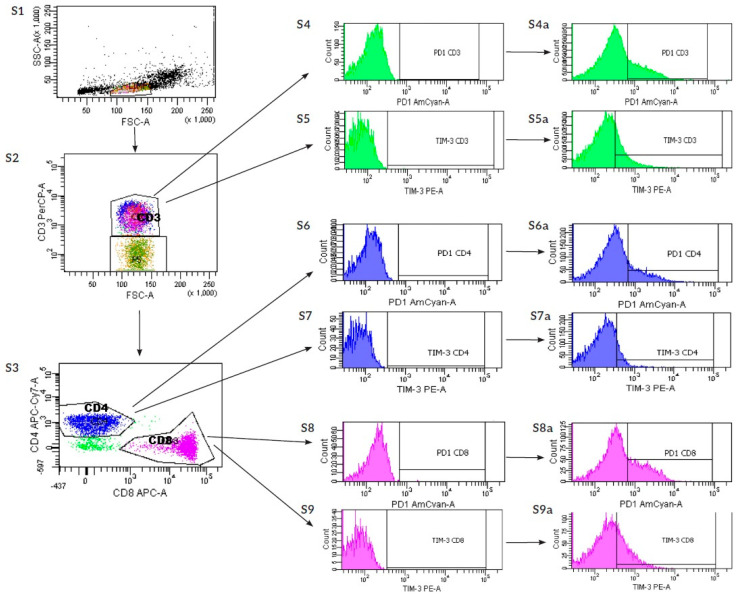
Lymphocyte T cytometric analysis scheme. The PE channel was used to label TIM-3 and TIGIT molecules, depending on the tube, and the AmCyan channel was used to label PD1, LAG-3, and VISTA molecules. (**S1**)—gate for lymphocytes based on SSC and FSC dot-plot, (**S2**)—CD3-PerCP fluorescence based T-cells gate, (**S3**)—CD4-APC-Cy7 and CD8-APC-A cells gates for T helper cells and cytotoxic T lymphocytes, (**S4**,**S5**)—histogram of FMO controls for TIM-3 and PD-1 on CD3 cells, (**S6**,**S7**)—FMO controls for TIM-3 and PD-1 on CD4 cells, (**S8**,**S9**)—FMO control for TIM-3 and PD-1 on CD8 cells, (**S4a**)—expression of TIM-3 on CD3 cells, (**S5a**)—expression of PD-1 on CD3 cells, (**S6a**)—expression of TIM-3 on CD4 cells, (**S7a**)—expression of PD-1 on CD4 cells, (**S8a**)—expression of TIM-3 on CD8 cells, (**S9a**)—expression of PD-1 on CD8 cells, S-sample, (**S1**–**S3**)—dot plots, and (**S4**–**S9**) a-histograms.

The cells from each version of the culture were collected from plates and centrifuged in PBS. The supernatant was gently discarded, and cells were suspended in 100 µL PBS with 0.01% sodium azide. The 1st step, staining with antibodies against surface antigens, was performed (Table 2). Prior to the experiment, each antibody was titrated to determine the concentration with the highest signal-to-noise ratio (Table 2). Cells were incubated with Ab for 15 min in the dark at room temperature. After the 1st step, staining, cells were washed in 1 mL of PBS (Corning, Glendale, AZ, USA) with 0.01% sodium azide NaN_3_ (Merck, Dramstadt, Germany) and centrifuged at 600× *g* for 10 min at room temperature. After washing, cells were resuspended in 400 µL 0.01% PBS with NaN_3_ and vortexed. Furthermore, cells were divided equally (250 µL) into 4 FACS tubes. The 1st tube served as FMO control; the 2nd tube was additionally stained with anti-PD-1 and anti-TIM-3 antibodies; the 3rd tube was stained with anti-LAG-3 antibodies; and the 4th tube was stained with anti-TIGIT and anti-VISTA antibodies (Table 3). Subsequently, cells were incubated for 15 min in the dark at room temperature. Finally, cells were washed with 1 mL of 0.01% NaN_3_ PBS and centrifuged at 600× *g* for 10 min at room temperature. After the washing step, cells were resuspended in 150 µL of 0.01%NaN_3_ PBS and prepared for acquisition. Flow cytometric analyses were performed using a BD FACSCanto™ II flow cytometer (BD Biosciences, San Jose, CA, USA) equipped with three lasers: blue (488 nm), red (633 nm), and violet (405 nm). Fluorescence signals were detected in the corresponding emission channels using optical filters of 530/30 nm, 585/42 nm, and 670 LP (for the 488 nm laser); 660/20 nm and 780/60 nm (for the 633 nm laser); and 450/50 nm, 525/50 nm, and 610/20 nm (for the 405 nm laser). Data analyses were performed using Diva 6.0 software. The scheme of flow cytometry analysis is shown in Figure 8.

### 4.6. Determination of Anti-HLA-Y Antibodies

To exclude alloimmunization, we determined anti-HLA-Y antibodies in the serum of all studied women. Anti-HLA-Y antibodies are directed against human leukocyte antigen Y (HLA-Y) and may reflect prior alloimmune sensitization after previous pregnancy, blood transfusion, or transplantation. Their presence has been associated with implantation failure, RPL, and pregnancy complications related to abnormal maternal immune activation [12,13]. Determination of anti-HLA-Y antibodies was performed with the Human anti-ATP-dependent RNA helicase DDX3Y antibody (Anti-DDX3Y Ab)—ELISA Kit, manufactured by Wuhan Enlibio Biotech Co., Ltd. (Wuhan, China). DDX3Y is one member of the DEAD box RNA helicases, encoded by the gene located on the chromosome Y in the azoospermia factor A region. Polyclonal anti-DDX3Y antibodies (14041-1-AP) are produced by immunizing animals with the C-terminus of DDX3Y and detecting a 73-kDa band.

The ELISA method was performed according to the manufacturer’s instructions. Pre-coated plate and compounds were equilibrated to room temperature. Standards, samples (100 µL) were added to wells in duplicate; wells containing 0 ng/mL received standard diluent. Plates were sealed and incubated at 37 °C for 90 min. After 2 washes, antigen (100 µL) was added to each well, incubated at 37 °C for 60 min, followed by 3 washes. Enzyme conjugate (100 µL) was added, incubated at 37 °C for 30 min, and washed 5 times. Color reagent (100 µL) was added, incubated in the dark until a visible gradient developed (≤30 min), followed by the addition of color reagent C (100 µL). Absorbance was read at 450 nm with a 630 nm correction filter within 10 min. The detection range was from 20 ng/mL to 0.312 ng/mL. The minimum detectable human anti-DDX3Y Ab was up to 0.06 ng/mL. Intra-assay precision ≤ 8%, and inter-assay precision ≤ 12%.

### 4.7. Institutional Review Board Statement

The study was conducted in accordance with the Declaration of Helsinki and approved by the Bioethics Committee of the Medical University of Warsaw. The Bioethics Committee approval number is KB/13/2020 and was issued on 19 January 2019.

### 4.8. Statistical Analysis

Based on the literature data indicating that the expression of immune-regulating molecules in the general population ranges from 40 to 60%, we assumed an average expression of Mi1 = 50%. A power analysis for the hypothesis that the mean expression Mi2 in women with recurrent miscarriages falls between 30 and 50% (Mi2 = 40%) was conducted. The alternative hypothesis assumed that Mi1 ≤ Mi2, with a significance level (α) of 0.05 and a standard deviation (σ) of 15%. For a predicted test power of 0.8, the required sample size (N) was calculated to be 20 individuals per group. Therefore, a sample size of 20 individuals per group was deemed justified, given the pilot nature of the project. Statistical analyses were performed using GraphPad Prism version 10.6.1 (GraphPad Software, San Diego, CA, USA). Data normality for each variable within each group was assessed using the Shapiro–Wilk test. Considering normal distribution, a mixed-effects model was used with repeated measures followed by Bonferroni’s multiple-comparison test for post hoc analysis. In cases of non-normal distribution, pairwise comparisons between time points were performed using the Wilcoxon matched-pairs signed rank test, with Bonferroni–Dunn’s method applied for multiple-comparison corrections. Corrections for multiple comparisons were applied consistently across all analyses, especially when multiple checkpoints and cell populations are compared simultaneously. Results were considered statistically significant at an adjusted *p*-value of <0.05. Data are presented as mean ± standard deviation.

### 4.9. Graphics

Graphics were created online with a free trial of BioRender.com, and modified with Microsoft Paint 3D for final image version. 

## 5. Conclusions

The observed, consistent modulation of immune checkpoint protein expression across NKT, NK, and regulatory T cells suggests that SH exert a systemic immunoregulatory effect rather than a cell type-restricted response. The concomitant upregulation of TIM-3, LAG-3, and PD-1 molecules associated with immune inhibition and tolerance, together with the downregulation of TIGIT, indicates a complex rebalancing of checkpoint pathways. In the context of recurrent pregnancy loss and unexplained infertility, such modulation may favor the establishment or maintenance of immune tolerance at the maternal–fetal interface. The uniformity of these effects across innate and regulatory lymphocyte subsets supports the hypothesis that SH influence shared signaling pathways involved in immune homeostasis. Although the functional consequences of these changes require further investigation, the present findings provide mechanistic insight into how SHs may contribute to immune regulation in disorders characterized by impaired reproductive immunotolerance.

## Figures and Tables

**Table 1:** Characteristics of the study participants, including age, BMI, miscarriage history, dietary supplement use, and chronic diseases. * *p* < 0.05, ** *p* < 0.005.

	Median and Q1–Q4 Quartile	Pregnant Women	RPL Women	*p*-Value Pregnant vs. RPL
Age [years]	Median	30	34	*p* = 0.26
	Q1	25	22	
	Q4	39	40	
BMI (body mass index)	Median	21.6	21.8	*p* = 0.5
	Q1	16.7	17.9	
	Q4	31.4	37.2	
Number of full-term pregnancies	Median	1	0	*p* = 0.027 *
	Q1	1	0	
	Q4	3	0	
Number of miscarriages	Median	0	3	*p* = 0.0001 **
	Q1	0	2	
	Q4	0	5	
Pregnancy duration (weeks)	Median	12.8	8.8	*p* = 0.1
	Q1	11.7	4.0	
	Q4	14.6	13.6	
The occurrence of chronic diseases				
		Pregnant women	RPL women	
Diabetes		10%	5%	
Endometriosis		0%	0%	
Insulin resistance		5%	5%	
Hashimoto’s disease		10%	20%	
Polycystic ovary syndrome		10%	0%	
Alloimmunization		5%	5%	
Diet supplements and folic acid administration before pregnancy and during pregnancy				
Folic acid administration		65%	80%	
Medicine and dietary supplement administration before pregnancy		60%	75%	
Salicylic acid and dietary supplement administration during pregnancy		90%	75%	
No medicine or dietary supplement administration before pregnancy		40%	25%	
No medicine or dietary supplement administration during pregnancy		10%	25%	

**Table 2:** Antibodies, clones, and titrated amounts, as well as the sources of antibodies used to label the main lymphocyte subsets, as FMO controls.

Antibody	Fluorochrome	Clone	Volume	Manufacturer
Anti-CD3	PreCP	Sk7	2 µL	Becton Dickinson, Franklin Lakes, NJ, USA
Anti-CD4	APC-Cy7	RPA-T4	0.5 µL	Becton Dickinson, Franklin Lakes, NJ, USA
Anti-CD8	APC	SK-1	0.5 µL	Becton Dickinson, Franklin Lakes, NJ, USA
Anti-CD25	FITC	CD25-4E3	1 µL	Becton Dickinson, Franklin Lakes, NJ, USA
Anti-CD127	BV450	HIL-7R-M21	0.5 µL	Becton Dickinson, Franklin Lakes, NJ, USA
Anti-CD56	PE-Cy7	B159	1 µL	Becton Dickinson, Franklin Lakes, NJ, USA

**Table 3:** Antibodies, clones, titrated amounts, and sources used for ICP staining.

Experiment	Antibody	Fluorochrome	Clone	Volume	Manufacturer
1st tube	-	-	-	-	-
2nd tube	Anti-PD1	BV480	EH12.1	1 µL	Becton Dickinson, Franklin Lakes, NJ, USA
	Anti-Tim3	PE	7D3	0.5 µL	Becton Dickinson, Franklin Lakes, NJ, USA
3rd tube	Anti-Lag3	BV480	T47-530	1 µL	Becton Dickinson, Franklin Lakes, NJ, USA
4th tube	Anti-TIGIT	PE	741182	1 µL	Becton Dickinson, Franklin Lakes, NJ, USA
	Anti-VISTA	BV480		1 µL	Becton Dickinson, Franklin Lakes, NJ, USA

## Data Availability

The raw data supporting the conclusions of this article will be made available by the authors on request.

## Supplementary Materials

The following supporting information can be downloaded at: https://www.mdpi.com/article/10.3390/ijms27031265/s1.

## Abbreviations

CTLA-4—cytotoxic T-cell antigen 4, DHT—dihydrotestosterone, P4—progesterone, E2—estradiol, FMO—fluorescence minus one, ICP—immune checkpoint proteins, LAG-3—lymphocyte-activation gene 3, IFN-γ—interferon-γ, MFI—maternal–fetal interface, NK cells—natural killer cells, PBMC—peripheral blood mononuclear cells, PBS—phosphate-buffered saline, PD-1—programmed cell death protein 1, PD-L1—programmed cell death ligand 1, PD-L2—programmed cell death ligand 2, RPL—recurrent pregnancy loss, Th1—T-helper lymphocyte 1, Th2—T-helper lymphocyte 2, TIGIT—T-cell immunoglobulin and ITIM domain, TIM-3—T-cell immunoglobulin-3, TNF-α—tumor necrosis factor-α, VISTA—V-domain Ig suppressor of T-cell activation.
