# Supplementary material for: Sex Hormones-Mediated Modulation of Immune Checkpoints in Pregnancy and Recurrent Pregnancy Loss
Source: Int J Mol Sci. 2026 Jan 27;27(3):1265. doi: 10.3390/ijms27031265 (PMC12898136; doi:10.3390/ijms27031265)
Supplement: Supplementary file 1 [file ijms-27-01265-s001.zip › ijms-4076928-supplementary.docx]

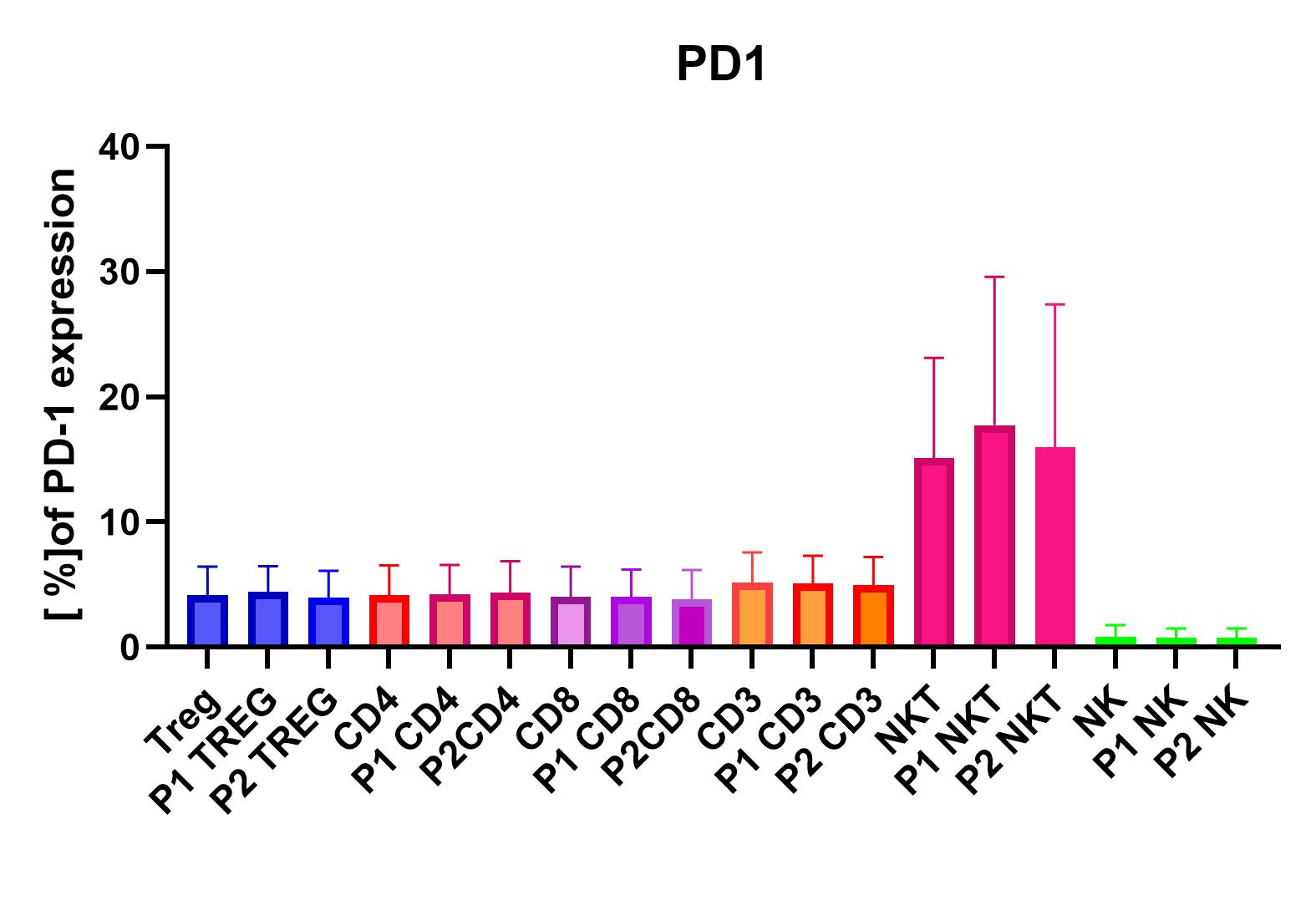


Fig.S1. E~~e~~ffect of progesterone at concentrations of P1 – 250 ng/ml and P2 – 500 ng/ml on PD-1 immune checkpoint (ICP) expression in immune cells subpopulations including Treg, CD4-Th, CD8-Tc, NKT and NK cells of RPL patients, Data are presented as the mean and SD of percentage of ICP-positive cells. Statistical significance: *p < 0.05, **p < 0.01, ***p < 0.001.


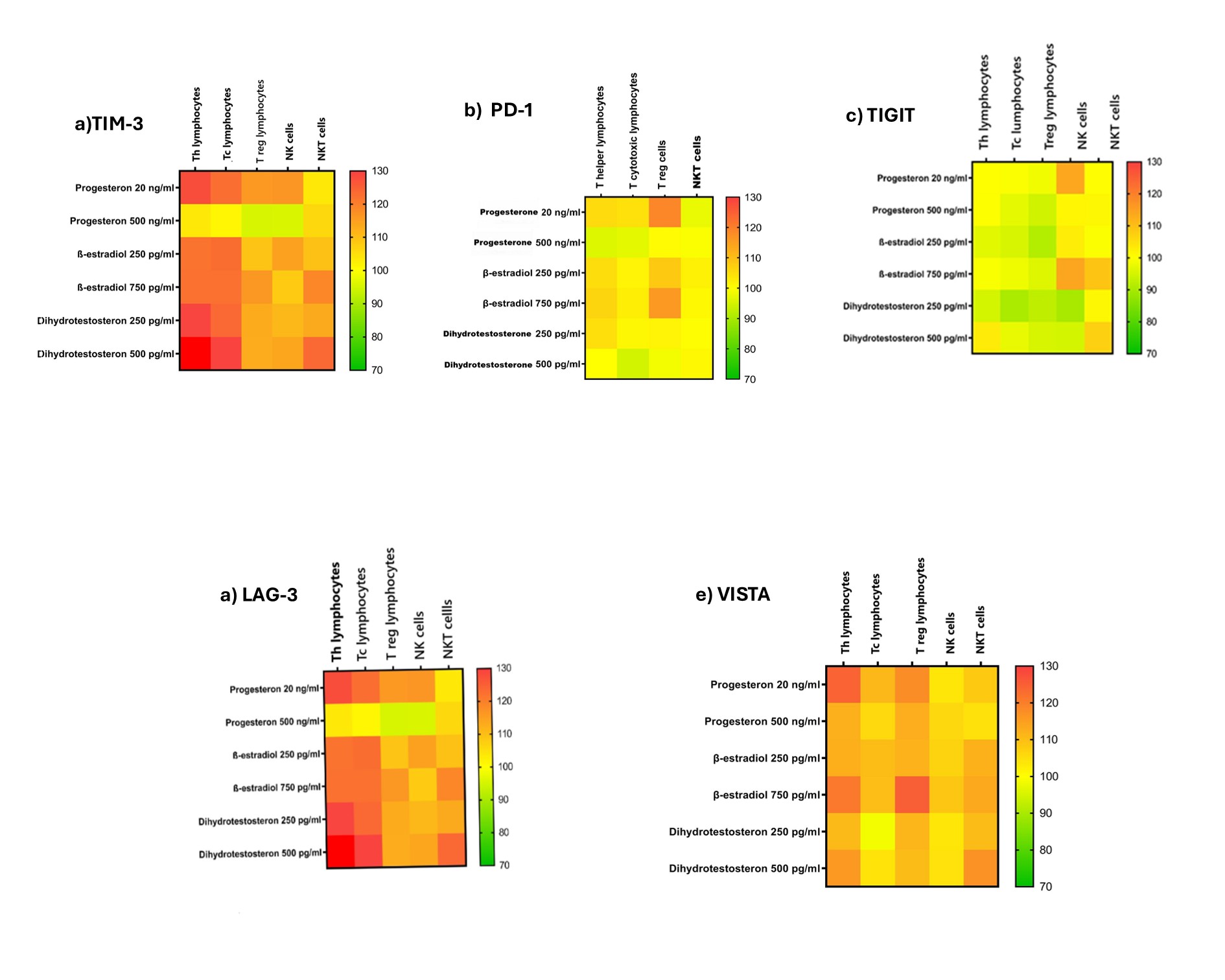


Fig.S2. Heat – maps of SH (progesterone, estradiol and dihydrotestosterone) influence on immune cells including Treg cells, Tc, Th, T, NK and NKT cells of pregnant women, n=20.


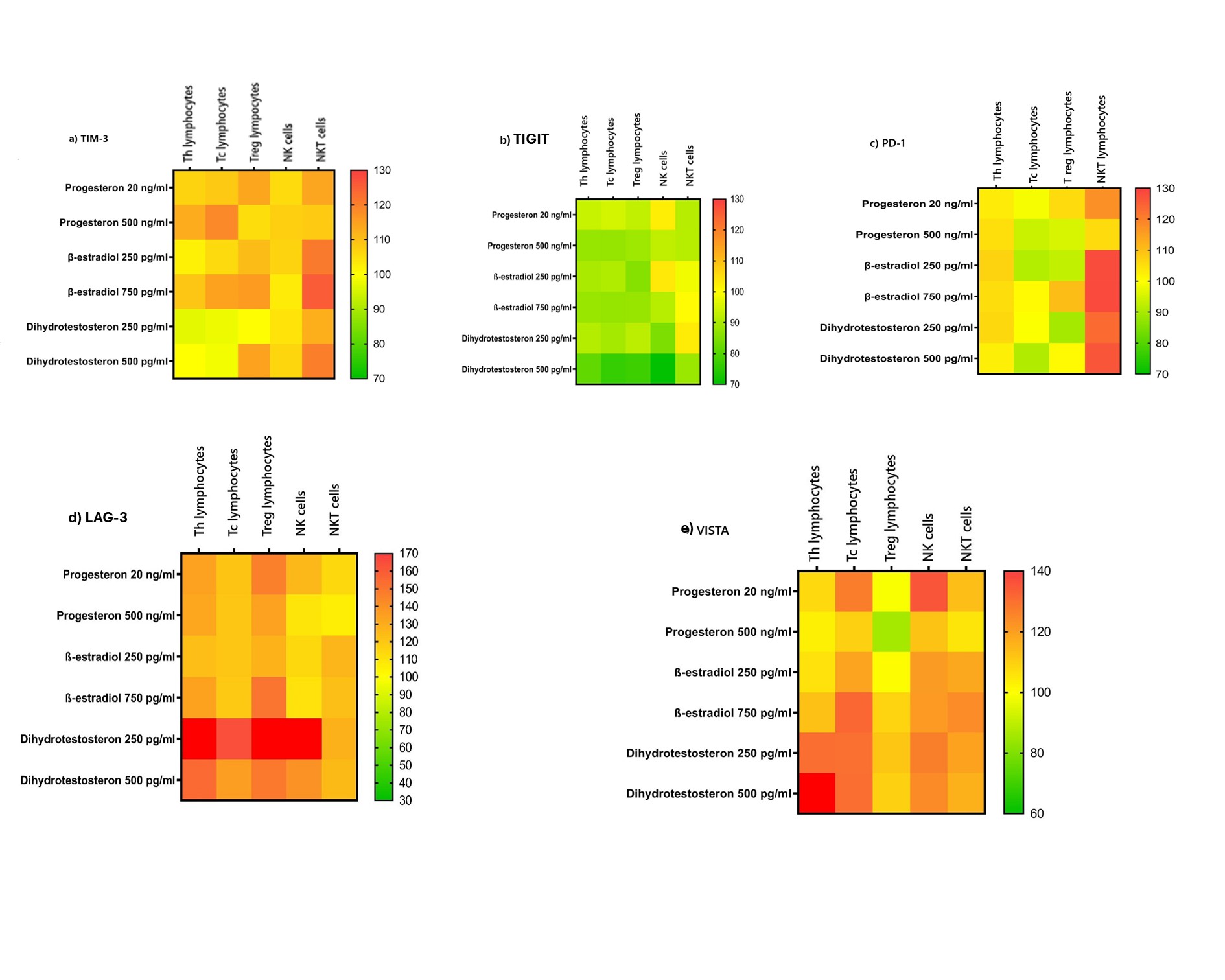


Fig.S3. Heat – maps of SH (progesterone, estradiol and dihydrotestosterone) influence on immune cells including Treg cells, Tc, Th, T, NK and NKT cells of uRPL group of women, n=20.
